# *Clostridioides difficile* infection in thoroughbred horses in Japan from 2010 to 2021

**DOI:** 10.1038/s41598-023-40157-x

**Published:** 2023-08-11

**Authors:** Eri Uchida-Fujii, Hidekazu Niwa, Mitsutoshi Senoh, Haru Kato, Yuta Kinoshita, Hiroshi Mita, Takanori Ueno

**Affiliations:** 1 Microbiology Division, Equine Research Institute, Japan Racing Association, Shiba 1400-4, Shimotsuke, Tochigi 329-0412 Japan; 2 Department of Bacteriology II, National Institute of Infectious Diseases, Gakuen 4-7-1, Musashimurayama, Tokyo 208-0011 Japan; 3 Antimicrobial Resistance Center, National Institute of Infectious Diseases, Aoba-Cho 4-2-1, Higashimurayama, Tokyo 189-0002 Japan; 4 Clinical Veterinary Medicine Division, Equine Research Institute, Japan Racing Association, Shiba 1400-4, Shimotsuke, Tochigi 329-0412 Japan

**Keywords:** Infection, Clostridium difficile

## Abstract

We encountered 34 *Clostridioides difficile* (*C. difficile*) infection (CDI) cases among Thoroughbred horses in Japan from 2010 to 2021. Among them, 79.4% (27/34) either died or were euthanised. The risk factors associated with CDI and mortality among Japanese Thoroughbred horses remain unclear. We used genetic methods to examine *C. difficile* strains and their relationships with prognosis. Twenty-two (64.7%) cases were hospitalised at the onset of colitis. Outcomes were balanced for hospitalisation rates at the onset of colitis. The mortality rates of cases treated with metronidazole (65.0%) were significantly lower than untreated cases (100%). The predominant genotype of *C. difficile* isolate was polymerase chain reaction ribotype (RT) 078, isolated from 12 cases (35.3%), followed by RT014 (six cases, 17.6%). Binary toxin (*C. difficile* transferase [CDT])-positive strains, including all RT078 strains, were isolated from 16 horses. Mortality rates in RT078 strain (75.0%) or CDT-positive strain (83.3%) cases were comparable to that in cases of other types. Sufficient infection control is needed to prevent CDI in Thoroughbred horses. A timely and prompt CDI diagnosis leading to metronidazole treatment would improve CDI outcomes.

## Introduction

*Clostridioides difficile* is a gram-positive, spore-forming bacterium considered one of the causative agents of colitis in humans, especially during antimicrobial treatment and hospitalisation^1^. Toxins produced by *C. difficile*, including toxin A, toxin B, and binary toxin (*C. difficile* transferase: CDT), are well-known virulence factors of *C. difficile*^2,3^. *C. difficile* has also been isolated from various animal species. Animals from which *C. difficile* is isolated are often asymptomatic but *C. difficile* also causes diseases in certain animal species such as horses and pigs^4^. In horses, *C. difficile* is one of the most important causes of colitis, and is pathogenic in foals and adults^5^.

*C. difficile* infections (CDIs) in horses have been reported worldwide. The clinical manifestations of CDI in horses vary from mild to severe^4^, and can be fatal in some cases. Arroyo et al. showed that the mortality rate was 37% in horses with toxigenic *C. difficile*^6^. Furthermore, Weese et al. reported deaths in 26% of CDI cases, even though the difference in mortality rate was not significant when CDI cases were compared with non-CDI colitis cases^7^. *C. difficile* has also been isolated from faecal samples, and 6–8% of healthy horses are thought to harbour *C. difficile*^8,9^. Treatment with antimicrobial agents is a risk factor for CDI in horses and humans^10^. Genotypes of some *C. difficile* isolated from horses, such as polymerase chain reaction (PCR) ribotypes (RT) 001, 012, 014, 017, and 0274, have been noted because they are isolated from both horses and humans. The RT078 strain, which is commonly isolated from pigs, and is known to be hypervirulent in humans^11,12^, has also been isolated from horses^8,13,14^.

Among Japanese Thoroughbred horses, the first CDI cases were observed in 2010 as healthcare-associated infections with RT078 *C. difficile*; Niwa et al. reported five fulminant CDI cases in Thoroughbred racehorses caused by RT078, which is considered as hyper-virulent ribotype in humans, in Japan^15^. This report described an outbreak of CDI in the equine hospital caused by a sole RT. Afterwards, approximately additional 20 cases of CDI with various clinical backgrounds in this population were analysed and reported by Nomura et al. from the clinical aspect^16^. Nomura et al. reported that CDI cases were not limited to hospitalised cases and > 80% of CDI cases were euthanised or died from 2010 to 2018^16^, and CDI in Japanese Thoroughbred horses was associated with a significantly increased mortality rate of colitis. Nomura et al. revealed that the treatment with metronidazole has decreased the mortality rate, although not significantly so^16^. However, the report lacked information on the genotype of *C. difficile* isolates, and clinical backgrounds of the cases, including those with/without hospitalisation at the onset of colitis signs have not been examined. This is needed to discuss risk factors for the poor prognosis of Japanese Thoroughbred horses with CDI. Analysis of *C. difficile* isolates could contribute to understanding the infection route and the risk factors related to the prognosis of Japanese Thoroughbred horses with CDI.

In this study, we performed molecular typing on *C. difficile* isolates from CDI cases in Japanese Thoroughbred horses to evaluate the pathogenicity of each genotype by linking the outcome. We also reviewed the clinical backgrounds of 34 CDI cases as well as described the genotypes of *C. difficile* isolates from CDI cases; the clinical findings of some cases were reported before^15,16^. Based on the obtained data, we discussed potential prognostic factors by examining the association between case characteristics and *C. difficile* genotypes.

## Results

### Background characteristics and prognostic outcomes

**Figure 1:**
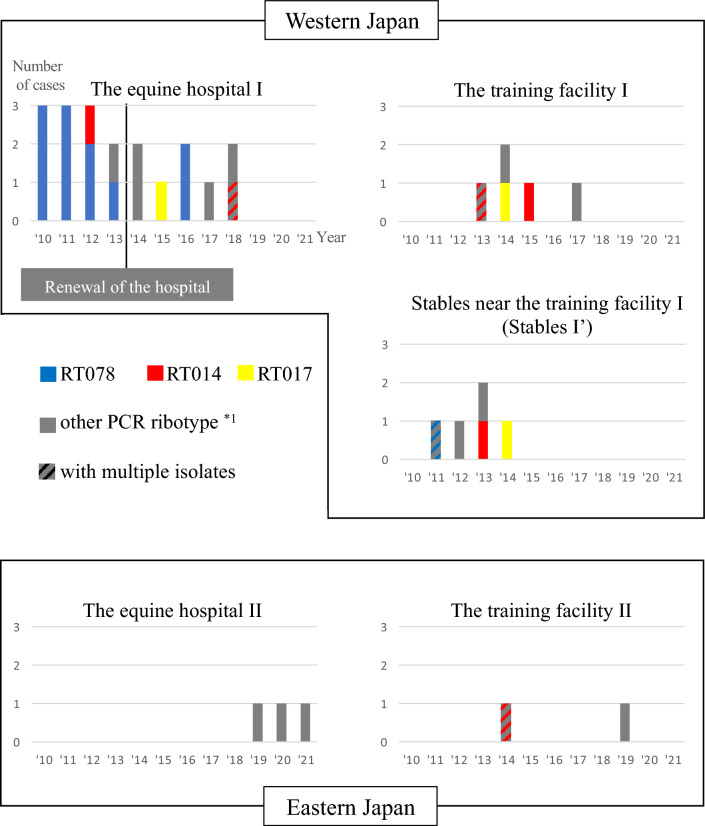
*Clostridioides difficile* infection cases and polymerase chain reaction ribotypes (RTs) of isolates during 2010–2021. The number of *Clostridioides difficile* infection cases and RTs of isolates for each year were shown by residence at the onset of colitis: equine hospital I, training facility I, stables near I (stables Iʹ), equine hospital II, and training facility II. Cases with multiple isolates of different RTs were shown as stripes. *1 RT c056, 056, 027, 002, hnc08162, j41, km0429, rh13124, 103, 046, rh2205, rh2208, y32, and rcc11084.

Table 1 shows the backgrounds and outcomes of the 34 Thoroughbred cases. Detailed information on each case is provided in Supplementary Table S1. The residence of cases shown in Table 1 and Fig. 1 indicated the location where the cases were kept at the onset of CDI. Moreover, 22 (64.7%) of the 34 cases were hospitalised in equine hospitals I and II due to treatment for other diseases and/or postoperative care at the onset of CDI. In addition, 25 cases (73.5%) were diagnosed during treatment for injuries or other diseases, whereas six cases (17.6%) presented immediately after transportation or while undergoing treatment for transport fever. As the onset of clinical signs, diarrhea, including watery diarrhea (26 cases, 76.5%) and bloody diarrhea (2 cases, 5.9%), was observed in all cases. In addition, 23 (67.6%) of the 34 cases showed abdominal pain. Twenty-six cases (76.5%) were under treatment with antimicrobials for other diseases at the onset of CDI, and five were not under antimicrobial treatment; the remaining three did not have sufficient information. Twenty cases (58.8%) received metronidazole to treat CDI. Of the 34 cases, 27 (79.4%) died or were euthanised 0–101 days (median 3 days) after the onset of the colitis, and only seven (20.6%) recovered (Table 1). Autopsies were performed on 24 cases. Gross pathological findings mainly included intestinal mucosal necrosis (21/24, 87.5%); pseudomembrane formation (2/24, 8.3%), catarrhal inflammation (1/24, 4.2%) and ulcers (1/24, 4.2%) in intestinal mucosa were also confirmed in some cases.

**Table 1:** The background characteristics and outcomes of *Clostridioides difficile* infection (CDI) among Japanese thoroughbred cases (N = 34).

Background characteristics	Number of cases	Percentage of the total number of cases (%)
Age (years)		
2–3	19	55.8
4–6	15	44.1
Sex		
Male	20	58.8
Female	9	26.5
Gelding	5	14.7
Residence of cases at the onset of CDI		
Equine hospital I	19	55.9
Training facility I	5	14.7
Stables near I (stables I′)	5	14.7
Equine hospital II	3	8.8
Training facility II	2	5.9
Comorbidities		
Surgery	19	55.9
Laparotomy	7	20.6
Orthopaedic surgery	6	17.6
Surgery for trauma	3	8.8
Other surgeries	3	8.8
Transportation/transport fever	6	17.6
Cellulitis	2	5.9
Other comorbidities	4	11.8
None	3	8.8
Antimicrobial use		
Yes	26	76.5
No	5	14.7
Unknown	3	8.8
Outcomes		
Death/euthanasia	27	79.4
Recovery	7	20.6

### Molecular analysis of *C. difficile* isolates

Thirty-eight *C. difficile* strains were isolated from the 34 cases, including a single strain from each of the 30 cases, and two strains with different RTs from each of the four cases (see Supplementary Table S1).

From 16 of the 34 cases, toxin A-positive, toxin B-positive, and CDT-positive (A^+^B^+^CDT^+^) strains were recovered (Table 2). The major RT was RT078, which was isolated from 12 of the 34 cases (35.3%). Nine cases were hospitalised at the equine hospital I from 2010 to 2013, two were hospitalised at the equine hospital I in 2016, and the remaining case was kept in stable Iʹ in 2011 when the horse showed colitis signs (Fig. 1). All 12 RT078 isolates were identified as 28–267 using PCR-based open-reading frame typing (POT). RT027 was isolated from one case in 2013 (Table 2).

**Table 2:** Risk factors for mortality in bivariable analysis (N = 34).

Variables (number of cases)	Outcome		Odds ratio (95% confidence interval)	*P* value
	Death or euthanasia (N = 27)	Recovery (N = 7)		
Hospitalisation at the onset of *Clostridioides difficile* infection				
Yes (22)	16 (59.3%)	6 (85.7%)	Reference	
No (12)	11 (40.7%)	1 (14.3%)	4.13 (0.43–39.21)	0.38
Treatments with metronidazole				
Yes (20)	13 (48.1%)	7 (100%)	Reference	
No (14)	14 (51.9%)	0 (0%)	Inf (1.22–Inf)	0.02
Toxin profile of *C. difficile*				
Toxin A-positive, toxin B-positive, binary toxin-positive (16)	12 (44.4%)	4 (57.1%)	0.6 (0.11–3.21)	0.55
Toxin A-positive, toxin B-positive, binary toxin-negative/toxin A-negative, toxin B-positive, binary toxin-negative (18)	15 (55.6%)	3 (42.9%)	Reference	
PCR ribotype (RT) of *C. difficile*				
RT078 (12)	11 (40.7%)	1 (14.3%)	4.13 (0.59–83.65)	0.17
Non-RT078 (22)	16 (59.3%)	6 (85.7%)	Reference	

Nineteen toxin A-positive, toxin B-positive, and CDT-negative (A^+^B^+^CDT^−^) strains were isolated from the 17 cases. Two A^+^B^+^CDT^−^ strains with different RTs were isolated from the two cases, and one A^+^B^+^CDT^−^ strain and one A^+^B^+^CDT^+^ strain were isolated from the two cases. In addition, six RT014 strains were obtained from six (17.6%) of the 34 cases. The occurrence of the cases was shown in both western and eastern Japan, with and without hospitalisation (Fig. 1). The results of the POT for RT014 strains were divided into five types: two isolates were 485–311, whereas the remaining four isolates differed from each other (306–323, 487–307, 359–279, 901–371).

Toxin A-negative, toxin B-positive, and CDT-negative isolates (A^−^B^+^CDT^−^) were isolated from three of the 34 cases (8.8%), and all three isolates were RT017, and were identified as 700–337, using POT. The three cases occurred in different locations at the time of colitis onset (Fig. 1).

### Risk factors for mortality

A bivariate analysis of the mortality rate with CDI using variables is shown in Table 2. There was no significant difference in mortality rates in terms of hospitalisation at the onset of CDI. For treatment, all 14 cases that did not receive metronidazole treatment died or were euthanised (100%). This was consistent with a significantly higher mortality rate compared to 13 of the 20 cases treated with metronidazole (65.0%). Furthermore, no significant differences in mortality rates were observed with respect to the isolation of the A^+^B^+^CDT^+^ and RT078 strains.

## Discussion

CDI in horses has been confirmed worldwide, and often occur among hospitalised cases or during the administration of antimicrobial agents^9,17^. In our study, the majority of cases were hospitalised and/or administered antimicrobials at the onset of CDI. Furthermore, almost 50% of cases showed colitis signs after surgery, and approximately 18% of cases showed signs immediately after transport, or while already undergoing treatment for transport fever. Surgery is considered a risk factor for CDI in Thoroughbred horses in Japan^16^, and transportation is a risk factor for CDI^18^. Consistent with this, our results also showed that transportation and surgery are risk factors for the development of CDI in horses, even though it was unclear how these factors contributed to the development of CDI in our sample. Since 2019, *C. difficile* infection has only occurred in the training facility II. The trend of CDI occurrence in each of the facilities drastically changed; however, there was no epidemiological event to explain the reason for this change; training facilities I and II were geographically distant (400 km apart) and the transfer of horses between the facilities was rare. Furthermore, the trends in the genetic characteristics of the isolates differed from each other; the RT078 strain, which accounted for the majority in equine hospital I, was not isolated in equine hospital II. It should be noted that the trend of isolated *C. difficile* changed after the renewal of equine hospital I in 2014. The outbreak due to the RT078 strain was observed in equine hospital I from 2010 to 2013, and the renewal of the hospital in 2014 might have stopped the outbreak. The renewed hospital had the stalls to isolate CDI cases and its structures were easier to disinfect than the former hospital, which should help prevent the outbreak of *C. difficile*.

In our sample, nearly 80% of CDI cases died or were euthanised. Among them, the majority (14/27) of cases were not treated with metronidazole. Veterinarians started to use metronidazole for CDI cases in 2013 in both equine hospitals I and II. According to a study conducted by Nomura et al., which reviewed the same group of CDI cases as the present study did, suggested that metronidazole treatment reduced the mortality rate of CDI cases, even though the number of cases was too low to obtain adequate statistical power^16^. In this study, the effect of metronidazole treatment on CDI was significant, with an increased number of cases. Treatment with metronidazole would be a major factor for recovery from CDI in these cases, even though the accumulation of experience of the veterinarians with CDI, the early diagnosis, and improvement of supportive care might also contribute to decreasing the mortality rate. The high mortality rate in CDI cases in Japanese Thoroughbred horses would be notable compared to that in former reports (26–37%)^6,7^, even though the reason is difficult to discuss due to the distinctiveness of Thoroughbred racehorses described below. All of our cases were under intensive training for racing or after participating race. For race, it has been reported that stresses might alter the immune system of Thoroughbred horses temporarily^19^ and might have negatively affected the immune systems in our cases. Protein-rich diets for racehorses may affect microbiota in their gut. More than half of our cases developed CDI after surgery with general anaesthesia. Cases that underwent laparotomy would have been in poor physical condition before the surgery, and even in cases that underwent orthopedic surgery, general anaesthesia would have temporarily affected their blood circulation.

Pathological findings also indicated the severity of conditions in our cases; mucosal necrosis was observed in most of the fatal cases. It was notable that pseudomembrane formation was observed in two cases; pseudomembranous colitis is known as the findings of CDI in humans^1^. In horses, it was reported that pseudomembrane formation was not specific in CDI and also observed in colitis with other pathogens in horses^20^.

In this study, the isolation of RT078- or CDT-positive strains did not affect the mortality of cases, which is consistent with a previous report that the genotypes or toxin gene profiles of *C. difficile* strains were not associated with the prognosis of CDI in horses6. Most of our Thoroughbred racehorses with CDI showed severe conditions and needed intensive care, regardless of the genotypes or toxin profiles of *C. difficile* isolates.

Among our *C. difficile* isolates, RT078 was the major RT. RT078 has been isolated from CDI horses^13^. In addition, RT078 strains have also been isolated from horses without gastrointestinal signs^14^. In our cases, RT078 was observed continuously in equine hospital I from 2010 to 2013, and isolates had the same results as POT and could be the same strains, including cases (cases No. 1 to 5 in this study) reported as nosocomial infections caused by the RT078 isolate in equine hospital I between 2010 and 2011^15^. Two cases with isolated RT078 occurred at Renewed Equine Hospital I in 2016, which was suspected to be a healthcare-associated infection based on the location and timing of the cases. The epidemiological relatedness between the two cases and nosocomial infection cases in 2010–2013 was unclear only with POT; therefore, further studies, using whole-genome sequencing, are warranted.

For strains other than RT078 during 2010–2013 and in 2016, healthcare-associated infections were not suspected. RT014 was isolated sporadically from six cases, and the transmission of the strains was excluded. Furthermore, the genotypes of RT014 isolates with POT differed from each other, suggesting a genetic variety of RT014 strains isolated in our cases. A more detailed examination of RT014 strains with whole-genome sequencing would provide genomic information on *C. difficile* isolates from horses.

RT078 was frequently isolated and could have been the cause of healthcare-associated infections in our cases, while RT078 has rarely been isolated from human CDI case in Japan^21^. RT078 might be more likely to cause healthcare-associated infections in Thoroughbred horses, unlike the pathology in Japanese people. RT027 has been reported as a highly virulent strain^22^, and caused outbreaks in humans in Europe and North America^23^, although it has rarely been isolated in Japan^21^. RT027 has been isolated from a case of equine colitis in the United States^24^. In our study, the RT027 isolate was obtained from only one case. Although RT027 is an uncommon strain in horses, monitoring the isolation of RT027 from horses is needed.

For some of the PCR ribotypes identified in this study, isolation from humans and suspected transmission between humans and animals were reported. RT014 was the major RT in this study, and is one of the major strains isolated from human cases in Japan^21^. A genomic study suggested the transmission of RT014 strains between pigs and humans in Australia^25^. RT017 strains were divided into two major lineages, and transmission of strains in one lineage between humans and animals was suggested^26^. In the United Kingdom, an increase in CDI cases occurring outside hospitals has been reported in humans^27^, and *C. difficile* isolation from animals should be considered as a potential carrier of the zoonotic pathogen. In Japan, the RT014 and RT017 strains from horses should be investigated for their relationship with human isolates.

Interestingly, some RTs differed in isolation status between humans in Japan and our cases. RT018, which shows A^+^B^+^CDT^−^ and has been the major strain in humans in Japan^21^, was not isolated in our cases. This trend is similar to a previous report in Italy, which indicated that RT018 was rarely isolated from animals, even though it is a major RT in humans in Italy^28^. RT369, which is dominant among A^–^B^+^CDT^−^ strains in Japan^21^, was not isolated in our cases. The differences in RTs of isolates might be due to differences in the infection source and intestinal environment of humans and horses, as well as differences in hospital treatment and hygiene regimes.

In this study, there is a potential limitation on diagnosis with bacterial culture and the number of the samples. The culture method might not detect bacteria that are difficult to grow on isolation media or unknown pathogenic bacteria which does not has appropriate media for isolation. It is possible that some cases in this study coincidently harbour *C. difficile* and developed colitis because of other reasons or pathogens; we did not detect toxins in faeces or intestinal contents but confirmed that *C. difficile* isolates were toxin-producing. However, all of our cases developed severe colitis, and some of them were supposed to be healthcare-associated cases with PCR ribotyping, which suggested that our cases suffered from colitis caused by *C. difficile*. In addition, the population of cases in this study was almost limited to Thoroughbred racehorses that we had access to in our hospital, which may have biased the background of the cases. Because of the limited number of CDI cases treated in this study, it is possible that the information obtained in this study is not representative of the information available for thoroughbred horses in Japan.

This report provides information on RTs of *C. difficile* isolates from Japanese horses, as well as we discussed the factors that can affect the prognosis of horse CDI in horses, including the background of the cases, treatment, and the genotype or toxin-producing type of *C. difficile*. Our study suggests that metronidazole treatment is needed for horses with adequately diagnosed CDI. In addition, monitoring the number of CDI cases and their prognosis in horses should be continued to establish better approaches for the treatment and prevention of CDI. Finally, to understand the transmission route of *C. difficile*, and to prevent healthcare-associated CDI infection in horses, strain-based surveillance for CDI should be continued. Genotyping for *C. difficile* of animal origin is important for understanding the presence and transmission of *C. difficile* among humans, animals, and the environment. Further investigations of CDI in horses might contribute to understanding its potential role as a zoonotic pathogen in terms of One Health.

## Methods

This study was approved by the Research Planning Committee of the Japan Racing Association (Approval Number: 2018-3263-05). Informed consent was obtained from horse owners by the attending veterinarian.

### Cases

In total, 34 cases of CDI among Thoroughbred horse cases from 2010 to 2021 (see Supplementary Table S1) were reviewed in this study. CDI is defined by veterinarians as follows: cases with diarrhea, fever (> 38.5 °C), and diagnosed as colitis, as well as cases positive for toxin-producing *C. difficile* culture, and negative for *Clostridium perfringens* and *Salmonella* species. All cases underwent symptomatic treatments such as supplemental fluids, anti-inflammatory drugs, and digestive medicine, among which, 20 were also administrated metronidazole.

The cases were either trained for a race at their residence, or were already hospitalised for other diseases at the onset of colitis signs. All cases were then hospitalised for treatment of CDI in equine hospitals I or II. Both hospitals for racehorses held 19 stalls for hospitalisation each, which were in large training facilities that kept approximately 2000 Thoroughbred racehorses in western (training facility I) and eastern (training facility II) Japan. In 2014, the building of equine hospital I was closed, and a new facility was constructed at adjacent locations due to dilapidation. The new facility held 23 stalls for the hospitalisation of horses. Despite occasionally competing on the racecourse outside the facilities, horses rarely moved between the two facilities. There were private stables near training facility I (stable Iʹ) and facility II (stable IIʹ). Each of the private stables keeps approximately 40–300 horses, and horses were transferred between stables Iʹ and training facility I or stables II’ and training facility II. Information on individual horses and their medical conditions was obtained from an in-house medical record system.

### Bacterial tests

Faecal samples, swab samples of the rectum or intestinal contents of each case (Supplementary Table S1) were subjected to bacterial isolation. Initially, the swab sample of the rectum was suspended in 500 µL sterile saline. To exclude bacteria other than spores, 100 µL of the suspension, faeces, or intestinal contents were mixed with an equal volume of 99% ethanol, and incubated for 1 h at room temperature. In addition, 100 µL of the incubated sample was cultured in a CCMA-EX plate (Nissui Pharmaceutical, Tokyo, Japan) under anaerobic conditions at 37 °C for 48 h. Isolated colonies were identified using the MALDI Biotyper CA 3.2 system (Bruker Japan, Kanagawa, Japan). Up to three *C. difficile* colonies for each sample were subcultured and subjected to PCR for toxin genes, PCR ribotyping, and POT.

Culturing for *C. perfringens* and *Salmonella* spp., which are known enteropathogenic bacteria for horses, was also conducted to confirm negative results for the species. The ethanol-treated samples were cultured on CW agar plates (Nissui Pharmaceutical) supplemented with 5% egg yolk under anaerobic conditions at 37 °C for 48 h for *C. perfringens*. The non-ethanol treated samples were cultured on a DHL agar plate (Nissui Pharmaceutical) under aerobic conditions at 37 °C for 24 h for *Salmonella* spp.

### Toxigenic and typing analysis of* C. difficile* isolates

The genomic DNA of *C. difficile* isolates was extracted using an InstaGene Matrix kit (Bio-Rad Japan, Tokyo, Japan) as per the manufacturer’s instructions. The presence of genes encoding toxin A, toxin B, and CDT was determined by PCR, as reported by Kato et al.^29,30^, and Stubbs et al.^31^. Briefly, the segment of the gene encoding toxin A and toxin B were amplified with two primer sets. One set included NK9 (5ʹ-CCA CCA GCT GCA GCC ATA-3ʹ), NK11 (5ʹ-TGA TGC TAA TAA TGA ATC TAA AAT GGT AAC-3ʹ), and NKV011 (5ʹ-TTT TGA TCC TAT AGA ATC TAA CTT AGT AAC-3ʹ), while the other set included NK104 (5ʹ-GTG TAG CAA TGA AAG TCC AAG TTT ACG C-3ʹ) and NK105 (5ʹ-CAC TTA GCT CTT TGA TTG CTG CAC CT-3ʹ). The PCR was performed under the following cycling conditions: 30 cycles at 95 °C for 20 s and 62 °C for 2 min. The gene encoding enzymatic of CDT was amplified with a primer set including cdtApos (5ʹ-TGA ACC TGG AAA AGG TGA TG-3ʹ) and cdtArev (5ʹ-AGG ATT ATT TAC TGG ACC ATT TG-3ʹ). The following thermal cycling conditions were used: 30 cycles at 95 °C for 45 s, 52 °C for 1 min, and 72 °C for 1 min and 20 s. The specific PCR products for these genes were confirmed by E-Gel Electrophoresis System (Life Technologies, Carlsbad, USA).

PCR ribotyping was performed in accordance with the modified Stubbs method^32^ described by Kato et al.^33^. Briefly, the PCR was performed with each primer 5ʹ-GTG CGG CTG GAT CAC CTC CT-3ʹ (positions at 1445 to 1466 of 16S rRNA gene) and 5ʹ-CCC TGC ACC CTT AAT AAC TTG ACC-3ʹ (positions at 20 to 1 of 23S rRNA gene) under the following conditions: 35 cycles at 95 °C for 20 s and 55 °C for 120 s, followed by incubation at 75 °C for 5 min. The PCR products were separated in 2.5% agarose gel at a constant voltage of 125 V for 3.5 h. UMCG12(3) strain and US42 (restriction endonuclease type BI/PFGE type NAP1/RT027)^34^ were used as reference strains for RT078 and RT027, respectively. When isolates harbouring different RTs were detected from a single sample, each type of isolate was considered an independent strain.

POT was performed using the Cica Genus C. diff POT kit (Kanto-Kagaku, Tokyo, Japan), which is a newly developed typing kit that uses multiple PCRs to detect the genomic variety of bacterial isolates^35^ and was used for typing *C. difficile* in accordance with the manufacturer’s instructions. The obtained band pattern of the 19 sets of PCRs was converted to two numerical values, i.e., POT1 and POT2, using a formula provided by the manufacturer.

### Statistical analysis

The associations between categorical variables were examined using the Chi-square or Fisher’s exact test, as appropriate. Statistical analyses were performed using JMP 16 software (SAS Institute Inc., Cary, NC, USA). A *P*-value < 0.05 was considered statistically significant.

### Supplementary Information

Supplementary Table S1.

## Data Availability

All data generated or analysed during this study are included in this published article and its Supplementary File.
