# Supplementary material for: Clostridioides difficile infection in thoroughbred horses in Japan from 2010 to 2021
Source: Sci Rep. 2023 Aug 11;13:13099. doi: 10.1038/s41598-023-40157-x (PMC10421859; doi:10.1038/s41598-023-40157-x)
Supplement: Supplementary file 1 — Supplementary Table S1. [file 41598_2023_40157_MOESM1_ESM.pdf]

Supplementary Table S1 Patients' backgrounds, prognosis, and information of *Clostridioides difficile* isolates for each case

		Patients' backgrounds										Characterisitcs of isolate			Reference[*4]
Case No.	Year	Age	Sex	Residence[*1]	Hospitalisation	Disease before on set of CDI	Used antimicrobial agents	Clinical signs	Treatment with metronidazole	Prognosis	Pathological findings of the organ from which *C. dificille* were isolated	Toxin producing[*3]	PCR ribotype	POT	
1	2010	4	male	I	+	laparotomy for colic	cephalotine	watery diarrhea	No	euthanasia	lung abscess inclding caseous necrosis	$A^+B^+CDT^+$	078	28-267	a, b
2	2010	6	female	I	+	screw fixation for bone fracture	cephalotine	bloodly diarrhea	No	death	necrosis of entire region of intestinal mucosa	$A^+B^+CDT^+$	078	28-267	a, b
3	2010	6	female	I	+	conjunctival flap	cephalotine, antimicrobial eyedrops (tobramycin)	watery diarrhea	No	euthanasia	necrosis of entire region of mucosa in large intestine	$A^+B^+CDT^+$	078	28-267	a, b
4	2011	3	female	I	+	screw fixation for bone fracture	cephalotine	bloodly diarrhea, abdominal pain	No	euthanasia	necrosis of broad region of caecal and colonic mucosa	$A^+B^+CDT^+$	078	28-267	a, b
5	2011	6	male	I	+	laparotomy for colic	cephalotine	watery diarrhea, abdominal pain	No	euthanasia	necrosis of entire region of intestinal mucosa	$A^+B^+CDT^+$	078	28-267	a, b
6	2011	2	male	I	+	laparotomy for colic	cephalotine	watery diarrhea, abdominal pain	No	death	necrosis of partial region of jejunal mucosa with pseudomembrane formation	$A^+B^+CDT^+$	078	28-267	a
7	2011	2	gelding	I'	-	castration	cefem antimicrobials	watery diarrhea, abdominal pain	No	death	necrosis of caecal and colonic mucosa	$A^+B^+CDT^-$<br>$A^+B^+CDT^+$	hnc08162<br>078	947-275<br>28-267	a
8	2012	3	male	I	+	experimental laparotomy	ND	watery diarrhea	No	euthanasia	necrosis of entire region of intestinal mucosa	$A^+B^+CDT^+$	078	28-267	c
9	2012	2	gelding	I	+	corneal ulcer	antimicrobial eyedrops (ofloxacine, tobramycine, cloramfenicol)	watery diarrhea, abdominal pain	No	euthanasia	necrosis and congestion of caecal and colonic mucosa	$A^+B^+CDT^-$	014	306-323	a
10	2012	4	male	I'	-	None	-[*2]	watery diarrhea, abdominal pain	No	euthanasia	necrosis of broad region of colonic mucosa	$A^+B^+CDT^-$	km0429	359-311	a
11	2012	2	male	I	+	laparotomy for colic	cephalotine	watery diarrhea	No	euthanasia	ND	$A^+B^+CDT^+$	078	28-267	a
12	2013	4	male	I	-	cellulitis	cephalotine, lalixine	watery diarrhea, abdominal pain	No	euthanasia	necrosis of entire region of intestinal mucosa	$A^+B^+CDT^-$<br>$A^+B^+CDT^+$	014<br>027	487-307<br>673-445	a
13	2013	3	male	I	+	arthroscopic surgery for bone fractures	kanamycin	watery diarrhea	Yes	recovery	NA	$A^+B^+CDT^+$	rh13124	672-191	a
14	2013	4	gelding	I'	-	transportation	-	watery diarrhea	Yes	recovery	NA	$A^+B^+CDT^-$	014	485-311	a
15	2013	6	female	I	+	laparotomy for colic	benzilpenicillin and streptomycin	diarrhea, abdominal pain	Yes	recovery	NA	$A^+B^+CDT^+$	078	28-267	a
16	2013	2	female	I'	-	transport fever	cefazorine	watery diarrhea, abdominal pain	Yes	euthanasia	necrosis of entire region of intestinal mucosa	$A^+B^+CDT^-$	c056	978-311	a
17	2014	3	male	II	-	cellulitis	cephalotine	watery diarrhea	No	euthanasia	catarrhal inflammation of small intestine and necrosis of ceacal and colonic mucosa	$A^+B^+CDT^-$<br>$A^+B^+CDT^-$	j41<br>014	359-339<br>359-279	a
18	2014	3	male	I	-	None	cephalotine	watery diarrhea, abdominal pain	Yes	euthanasia	haemorragic necrosis of entire region of intestinal mucosa	$A^+B^+CDT^-$	056	978-55	a
19	2014	6	male	I	-	pneumonia	cephalotine, minocycline	watery diarrhea, abdominal pain	Yes	euthanasia	necrosis of ceacal and colonic mucosa	$A^-B^+CDT^-$	017	700-337	a
20	2014	2	male	I'	-	transportation	-	watery diarrhea, abdominal pain	Yes	euthanasia	necrosis of ceacal and colonic mucosa	$A^-B^+CDT^-$	017	700-337	a
21	2014	5	male	I	+	colic	-	watery diarrhea, abdominal pain	Yes	death	necrosis of ceacal ans colonic mucosa and peritonitis	$A^+B^+CDT^-$	c056	978-275	a
22	2014	6	female	I	+	surgery for trauma	cephalotine	watery diarrhea	Yes	death	necrosis of ceacal and colonic mucosa	$A^+B^+CDT^-$	002	954-375	a
23	2015	4	male	I	+	transport fever	cephalotine, fosphomycine, metronidazole	diarrhea	Yes	euthanasia	multifocal necrosis of colonic membrane with pseudomembrane formation	$A^-B^+CDT^-$	017	700-337	c
24	2015	3	male	I	-	None	-	watery diarrhea, abdominal pain	Yes	euthanasia	ulcer and erosion of colonic membrane and congestion of lamina propria	$A^+B^+CDT^-$	014	485-311	a
25	2016	5	male	I	+	transport fever	cephalotine, minocycline	watery diarrhea	Yes	euthanasia	necrosis of entire region of intestinal mucosa	$A^+B^+CDT^+$	078	28-267	a
26	2016	3	male	I	+	surgery of the laryngeal region	cephalotine, minocycline	watery diarrhea, abdominal pain	Yes	euthanasia	ND	$A^+B^+CDT^+$	078	28-267	a
27	2017	4	gelding	I	+	laparotomy for colic	cephalotine, polymyxin B	diarrhea, abdominal pain	No	euthanasia	necrosis and congestion of entire region of intestinal mucosa	$A^+B^+CDT^-$	103	947-311	c
28	2017	2	male	I	-	diarrhea and uveitis	ND	diarrhea, abdominal pain	Yes	death	acute peritonitis and edema of ceacal and colonic mucosa	$A^+B^+CDT^-$	y32	786-279	c
29	2018	4	gelding	I	+	surgery for trauma	cephalotine, minocycline	diarrhea, abdominal pain	Yes	recovery	NA	$A^+B^+CDT^-$	rh2205	1013-439	c
30	2018	3	female	I	+	screw fixation for bone fracture	cephalotine	diarrhea, abdominal pain	No	euthanasia	necrosis of entire region of intestinal mucosa	$A^+B^+CDT^-$<br>$A^+B^+CDT^-$	046<br>014	487-307<br>901-371	c
31	2019	3	male	II	+	surgery for trauma	cephalotine	watery diarrhea, abdominal pain	Yes	recovery	NA	$A^+B^+CDT^-$	rh2208	1019-311	c
32	2019	2	male	II	-	transportation	ND	watery diarrhea, abdominal pain	Yes	euthanasia	ND	$A^+B^+CDT^-$	103	947-311	c
33	2020	2	female	II	+	screw fixation for bone fracture	cephalotine	watery diarrhea, abdominal pain	Yes	recovery	NA	$A^+B^+CDT^+$	rcc11084	68-27	c
34	2021	3	female	II	+	screw fixation for bone fracture	cephalotine	watery diarrhea, abdominal pain	Yes	recovery	NA	$A^+B^+CDT^+$	rcc11084	68-283	c

ND: no data, NA: not applicable

*1 I: training facility in western Japan (the training facility I), I': stable near I (stables I'), II: trainig facility in eastern Japan (the training facility II)

*2 Antimicrobials were not administrated.

*3 $A^+B^+CDT^+$, toxin A-positive, toxin B-positive, binary toxin-positive; $A^+B^+CDT^-$, toxin A-positive, toxin B-positive, binary toxin-negative; $A^-B^+CDT^-$, toxin A-negative, toxin B-positive, binary toxin-negative

*4 a: Cases included in previous reports (Nomura et al, 2020) except for information of isolates

b: Cases included in previous reports (Niwa et al, 2013) except for the result of POT

c: Cases described first in this study
